# Characterization of a *DRC1* null variant associated with primary ciliary dyskinesia and female infertility

**DOI:** 10.1007/s10815-023-02755-6

**Published:** 2023-03-01

**Authors:** R. Pereira, V. Carvalho, C. Dias, T. Barbosa, J. Oliveira, Â. Alves, E. Oliveira, R. Sá, M. Sousa

**Affiliations:** 1 Laboratory of Cell Biology, Department of Microscopy, ICBAS-School of Medicine and Biomedical Sciences, University of Porto, Rua Jorge Viterbo Ferreira, 228, 4050-313 Porto, Portugal; 2 UMIB-Unit for Multidisciplinary Research in Biomedicine, ICBAS-UP/ ITR-Laboratory for Integrative and Translational Research in Population Health, University of Porto, Porto, Portugal; 3 Department of Biology, Faculty of Sciences, University of Porto (FCUP), Porto, Portugal; 4 Department of Pneumology, Hospital de Santo António (HSA), Centro Hospitalar do Porto (CHUPorto), Porto, Portugal; 5 Department of Children and Adolescents, Centro Materno-Infantil do Norte (CMIN), Centro Hospitalar Universitário do Porto (CHUPorto), Porto, Portugal; 6 Center for Predictive and Preventive Genetics, Institute of Health Research and Innovation (IBMC/i3S), University of Porto, Porto, Portugal

**Keywords:** Primary ciliary dyskinesia, Female infertility, *DRC1*, Twin sister, Recurrent otitis media, Cystic bronchiectasis

## Abstract

**Propose:**

We here present a female case with primary ciliary dyskinesia (PCD) and infertility. In this report, we also present the evaluation of the patient family, including her twin sister, also with PCD and infertility.

**Methods:**

Confirmation of the PCD clinical diagnosis was performed through assessment of cilia motility, by high-speed video microscopy (HSVM), axoneme ultrastructure, by transmission electron microscopy (TEM), and genetic characterization, by whole-exome sequence (WES). Gene expression studies used qPCR for mRNA expression and immunofluorescence to determine cell protein localization.

**Results:**

We identified a homozygous nonsense variant in the *DRC1* gene (NM 145038.5:c.352C>T (p.Gln118Ter)) in the female patient with PCD and infertility that fit the model of autosomal recessive genetic transmission. This variant eventually results in a dyskinetic ciliary beat with a lower frequency and a partial lack of both dynein arms as revealed by TEM analysis. Moreover, this variant implies a decrease in the expression of *DRC1* mRNA and protein. Additionally, expression analysis suggested that *DRC1* may interact with other DRC elements.

**Conclusions:**

Our findings suggest that the *DRC1* null variant leads to PCD associated with infertility, likely caused by defects in axoneme from Fallopian tube cilia. Overall, our outcomes contribute to a better understanding of the genetic factors involved in the pathophysiology of PCD and infertility, and they highlight the interaction of different genes in the patient phenotype, which should be investigated further because it may explain the high heterogeneity observed in PCD patients.

**Supplementary Information:**

The online version contains supplementary material available at 10.1007/s10815-023-02755-6.

## Introduction

Primary ciliary dyskinesia (PCD, ORPHA: 244) is predominantly a rare autosomal recessive disease, with some rare cases of X-linked [1, 2] and one gene proposed to be autosomal dominant (FOXJ1-PCD) [3, 4]. PCD has an estimated prevalence of 1:10,000 to 1:20,000 [5]; however, a recent large data analysis revealed that PCD is more common than previously thought, with a prevalence of 1:7500 globally [6].

PCD is a highly complex and heterogeneous disease, both genetically and phenotypically, caused by a dysfunction in the structure and/or function of motile cilia [7]. The axoneme is the ciliary motor, being composed by two central single microtubules surrounded by nine peripheral doublet microtubules, forming a cylinder with a 9d+2s pattern. Each doublet consists of an internal complete microtubule A, composed by 13 protofilaments, onto which is attached a second external and incomplete microtubule B, composed by 10 protofilaments [8, 9]. From microtubule A emerge two dynein arms (DA), the outer dynein arm (ODA) and the inner dynein arm (IDA). Doublets are interlinked by a nexin-dynein regulatory complex (N-DRC, previously known as nexin bridges) and are connected to the two central microtubules by radial spokes (RS) [9]. The central pair is connected by a central bridge and surrounded by a central sheath, constituting the central pair complex (CPC). The N-DRC is known to be involved in interdoublet sliding and signal mediation between different axoneme components, thus functioning as intermediary in the signaling pathway between the CPC-RS complex and the DA [10].

PCD includes many life-threatening symptoms that should not be neglected and, despite being a rare disease, symptoms are quite common, such as upper and lower respiratory tract complications, including neonatal intensive care admittance, chronic rhinosinusitis, hearing impairment, chronic bronchitis, bronchiectasis, and congenital cardiac defects [11]. Laterality defects (or *situs inversus)* are often observed among patients with PCD, with about 50% of patients showing Kartagener syndrome (triad of situs inversus, chronic sinusitis, and bronchiectasis) (ORPHA: 101063). Lastly, subfertility or infertility is also a feature of PCD [12, 13]. Most men with PCD show total/partial sperm immotility, caused by structural or functional deficiencies of the sperm flagellum axoneme, which has the same structure of cilia axonemes [12]. Some patients may also present azoospermia due to a dysfunction in cilia present in the *rete testis* and efferent ducts, which consequently impairs sperm transport to the epididymis [14, 15].

Regarding the fertility of women with PCD, the mechanisms and its prevalence are still unclear, but generally, women are less affected than men with PCD. In the female reproductive tract, ciliated cells found in the fallopian tube are identical to respiratory cilia both in terms of composition and in the beating pattern and frequency [16]. In the fallopian tube, the ciliary beat frequency was proposed to be regulated by progesterone [17]. The coordinated beating of fimbria cilia generates a directed fluid flow toward the uterine cavity, which intrinsically interacts with muscle contractions, to allow the propulsion of the embryo, likely causing an anomalous embryo transport toward the uterine cavity [18]. Therefore, in women, dysfunction of motile cilia in the fallopian tube is, so far, the most plausible explanation for infertility [18]. Some authors suggested that cilia dysfunction in the fallopian tubes of women with PCD is associated with an increased risk of ectopic pregnancy and pregnancy loss [13, 19, 20]. In those cases, to achieve pregnancy, assisted reproductive techniques are needed [21]. Nevertheless, some female patients with PCD can spontaneously conceive despite presenting severely dysfunctional cilia [13, 16]. To justify this dichotomy observed in female fertility, it was proposed that motile cilia dysfunction in fallopian tubes may be compensated by other mechanisms, such as muscle contractions and peristaltic movements, thus reducing the incidence of female infertility [22]. Besides, the female genotype might also be a justification for this, with some genes being proposed to be associated with female infertility in PCD patients, namely *CCDC39*, *CCDC40*, *DNAAF1*, and *LRRC6* [13, 20]. DRC1 is a highly conserved structural component of the N-DRC. It was proposed to function in the assembly and regulation of specific classes of IDA arm motors and to participate in the generation of ciliary bending [23]. The N-DRC is a complex structure that is anchored to microtubule A of peripheral doublets near the RS linkage. The N-DRC is structurally composed by the base plate, which is attached to the bottom of microtubule A, and the linker, which extends from microtubule A toward microtubule B of the neighboring peripheral doublet [10]. The DRC1 protein (also known as CCDC164) is one of the eleven subunits known to compose the N-DRC base plate (together with DRC2, also known as CCDC65) functioning as a scaffold and playing a pivotal role in the assembly of the entire N-DRC structure [23–26].

Here, we report a comprehensive clinical, functional, ultrastructural, and genetic characterization of a woman with clinical features compatible with PCD. Whole-exome sequencing (WES) identified a null homozygous variant in the *DRC1* gene. This was followed by additional mRNA and protein expression studies, which further corroborated the pathogenicity of this variant. In addition, we showed evidence pointing to gene interactions between *DRC1*, *CCDC65* (previously known as *DRC2*), and *CCDC40* in the patient phenotype. Additionally, all family members were evaluated, with the same variant found. As the patient and her twin sister both present idiopathic infertility and the same homozygous variant, in the absence of other infertility factors, it is plausible to raise the suspicion that in both cases the cause of infertility may lie in disruptive cilia motility/function in the fallopian tubes.

## Material and methods

### Ethics

All ethical guidelines were followed, with clinical data and biological material obtained under strict individual anonymity and after patient written informed consent. This work did not involve human or animal experiments and thus the provisions of the Declaration of Helsinki as revised in Tokyo 2004 do not apply to this work. Reproductive clinical data were obtained according to the provisions of the National Law on Medically Assisted Procreation (Law 32/2006) and the National Council for Medically Assisted Procreation guidelines (2018). Patient clinical data were obtained according to the hospital regular assessment guidelines for patients, under written informed consent. Biological material from the patient, family members and controls, was obtained after written informed consent and used in experiments according to the Joint Ethics Committee of the Hospital and University, CHUP/ICBAS approval number 2020-094 (077-DEFI-078-CE).

### Patient data

**Fig. 1:**
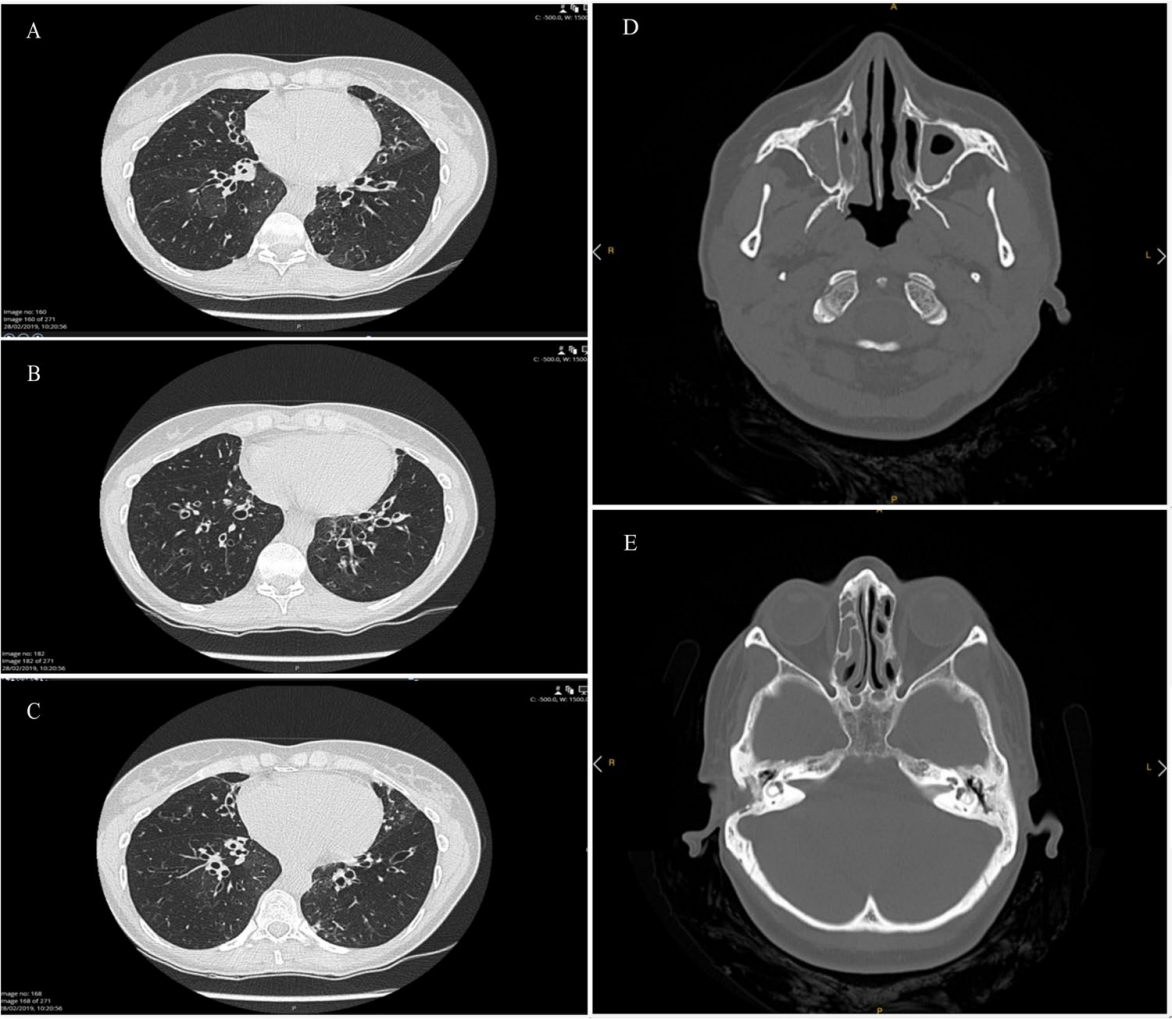
CT scan of the proband. **A**-**C** Thorax axial CT scan of the proband. **A** Note the presence of cystic bronchiectasis in the middle lobe and lower lobes. **B** and **C**. Note the presence of cystic bronchiectasis in the lower lobes. **D** Axial CT scan of paranasal sinuses showing obliteration of the maxillary sinus (complete in the right, partial in the left). **E** Axial CT scan shows opacification of the ethmoidal air cells bilaterally, more pronounced at the right. Right mastoidectomy

The female patient with PCD included in this study (proband) presented, since infancy, recurrent otitis media with bilateral hearing deficit, and progressively developed rhinosinusitis, persistent cough, sometimes with purulent sputum, and respiratory recurrent infections. She did not present dyspnea, wheezing, hemoptysis, weight loss, asthenia, fever, or gastrointestinal symptoms. She was submitted to nasosinusal endoscopic surgery and ear surgery. The patient is a non-smoker and her marriage is not consanguineous. The high-resolution thorax computed tomography (CT) scan showed cystic bronchiectasis with lower lobe predominance, with absence of laterality defects (Fig. 1). She performed respiratory function tests, showing a severe obstructive ventilatory defect (percent predicted value of FEV1 = 40%) (FEV1 = forced expiratory volume at the end of the first second of forced expiration). The blood count was normal (without peripheral eosinophilia), and the serologic Phadiotop evaluation was negative. Serum immunoglobulin levels (IgA, IgG, IgM, and IgE), serum IgG subclass titers, and the alpha-1-antitrypsin (AAT) serum concentrations were normal. The serum levels of *Aspergillus fumigatus*-specific IgE and IgG were normal. The bronchodilation test was negative. As the sweat chloride concentration result was borderline (30 mmol/L), it was performed an extensive genotyping of the complete *CFTR* gene for cystic fibrosis (sequencing of all *CFTR* exonic regions and exon-intron junctions), which identified a heterozygous pathogenic variant in exon 16, c.617T>G, P.Leuc206Trp. It was not detected deletions/duplications (by the MLPA technique). Repeated sputum cultures evidenced chronic bronchopulmonary colonization with *Pseudomonas aeruginosa*. Chronic treatments include inhaled antibiotics for infections, nebulization with hypertonic saline, bronchodilators, kinesiotherapy, and respiratory exercises to help clearance of the bronchial mucus.

The husband of the proband presents a normal karyotype (46, XY) and the spermiogram evaluation was normal according to reference values [27]. He is a non-smoker and do not present respiratory complains. After 2 years of attempts at natural conception, the couple began infertility consultations. The patient had regular menstrual cycles, normal hormone levels, normal antral follicle count, patent fallopian tubes (i.e., fallopian tubes not blocked), normal uterine cavity, and a normal karyotype (46, XX). The couple was diagnosed with idiopathic infertility. After two unsuccessful intrauterine insemination cycles, they performed an in vitro fertilization (IVF) cycle, with transfer of two embryos at day 3 [28]. The patient attained a successful twin pregnancy, with cesarean delivery, at 37 weeks of gestation, of two female newborns, with 2840 g and 2210 g [29], and an Apgar score of 8/9 and 9/10. The children are currently healthy, without respiratory complains.

The father of the proband presents allergies to pollen and grasses, and asthma. The mother of the proband presents bronchiectasis. Both parents are smokers, and their marriage is not consanguineous.

The proband has a twin sister, whose marriage is also not consanguineous. Since infancy, she presented atopic allergy, recurrent otitis media, and rhinosinusitis, with ear surgery and a 1-month hospitalization episode due to massive hemoptysis triggered by cough. She is a non-smoker. A high-resolution thorax CT scan showed discrete bronchiectasis in the middle lobar bronchus, without laterality defects. The respiratory function tests evidenced a mixed ventilatory alteration with moderate obstructive component (percent predicted value of FEV1 = 63%), decreased alveolar-capillary transfer capacity of carbon monoxide, with normalization after correction by alveolar volume. She also performed the same exclusion tests above mentioned for her sister (the proband), with the same normal results. Her husband is a smoker and presents allergies to pollen and grasses. After 7 years of attempts at natural conception, the couple went to infertility consultations, where idiopathic infertility was diagnosed. They performed an IVF cycle, with transfer of two embryos at day 2. She got a successful single pregnancy, with vaginal delivery, at 39 weeks of gestation, of one female newborn, with 3290 g and an Apgar score of 9/10. The child is currently healthy, without respiratory complains.

### Sample collection

Patient (proband) peripheral blood used for DNA extraction was collected in EDTA tubes (VACUETTE, Porto, Portugal). Sputum from the parents, twin sister, and children, used for DNA extraction, was collected with the Oragene DNA OG-500 kit (DNA Genotek Inc., Ontario, Canada).

Nasal cells were obtained by nasal brushing from the proband, parents, and healthy controls (7 individuals), using a cytology soft sterile brush (Endobrush, Biogyn SNC, Mirandola, Italy), in both nostrils. After brushing, cells of one nostril were placed in Medium 199 (Gibco, ThermoFisher Scientific, USA), supplemented with 50 μg/mL PenStrep, for high-speed video microscopy (HSVM) analysis, RNA analysis, and immunofluorescence. Cells from the other nostril were placed in fixative (2.5% glutaraldehyde in cacodylate buffer 0.1 M) for transmission electron microscopy (TEM) analysis. Control nasal cells obtained from healthy volunteers were used for RNA analysis and immunofluorescence.

### Transmission electron microscopy

Nasal samples of the proband were analyzed as previously reported [12]. Briefly, samples were fixed with 2.5% glutaraldehyde (Sigma-Aldrich, Missouri, USA) in 0.1 M cacodylate buffer (Merck, Darmstadt, Germany), pH 7.2, 2h, room temperature (RT), post-fixed with 2% osmium tetroxide (Merck) in buffer, 2h, 4°C, and dehydrated in a graded ethanol series (VWR, Pennsylvania, USA), then treated with 1% tannic acid (Merck) in 100% ethanol and embedded in epoxy resin (Epon, Sigma-Aldrich). Semithin and ultrathin sections were cut on an LKB-ultramicrotome (Leica Microsystems, Wetzlar, Germany), using diamond knives (Diatome, Pennsylvania, USA). Suitable areas of ciliated cells were selected in semithin sections (1 μm), and stained with methylene blue-Azur II (Merck). Ultrathin sections were retrieved on copper grids (Taab, Berks, England). After double-contrasting with aqueous uranyl acetate (BDH, Poole, England) and lead citrate (Merck), they were observed and photographed on a JEOL 100CXII transmission electron microscope (JEOL, Tokyo, Japan), operated at 60 kV.

### Quantitative TEM morphologic analysis

Cilia axoneme ultrastructure was evaluated at high magnifications in transverse sections. Diagnosis was based on the presence of a systematic defect in any of the axonemal structures [30], according to the quantitative methods provided by the international consensus guideline for reporting transmission electron microscopy results in the diagnosis of PCD (BEAT PCD TEM Criteria) [31].

### Quantitative TEM ciliary beat axis analysis

The ciliary beat axis and the ciliary deviation were evaluated in a minimum of 100 transverse sections examined after printing. In the printed images, a line was drawn parallel to the central microtubules. Based on the main orientation of the drawn lines, a perpendicular reference line was then chosen and the angle of each line parallel to the reference line was calculated and subtracted from the mean, with the differences being close to zero. The standard deviation (SD) of these differences corresponds to the ciliary beat axis and ciliary deviation [32].

### Ciliary beat frequency and beat patterns by high-speed video microscopy

Ciliary beat frequency (CBF) and ciliary beat patterns (CBP) were evaluated as previously described, with some adaptations [33]. Briefly, collected ciliated cells were placed in Medium 199, and immediately upon arrival at the laboratory, placed at 37°C until analysis. Analysis was conducted in an inverted microscope (Olympus, Nikon, Tokyo, Japan). Undisrupted ciliated clusters devoid of mucus were selected for study. Beating ciliated cells were recorded using a digital high-speed video camera (Semiconductor Vita 5000, Pixelink/Navitar, Inc., New York, USA) at a rate of 300 to 400 frames per second.

The CBF was analyzed in the proband and her parents, by two methods, the manual method using ImageJ, version 1.53e [34] and the semi-automated method with the CiliarMove program [35]. For manual analysis, it was registered the number of frames required to complete 10 cycles, which were then converted to CBF using the calculation (CBF = total number of frames/(number frames for 10 beats) × 10). In total, 33 and 42 ciliary regions from the parents and probands, respectively, were examined using the two techniques.

To assess CBP, each edge was analyzed using Image J. Coordinated ciliary beat in a back-and-forth movement along the entire epithelial edge was defined as normal. The dyskinetic beat pattern was categorized into eight distinct CBPs by a modification of previously reported descriptions (immotile, circular, hyperkinetic, hyperkinetic with reduced beat amplitude, asynchronous, asynchronous with reduced beat amplitude, stiff, and synchronous with reduced beat amplitude) [36, 37].

### Nucleic acid extraction

Genomic DNA was extracted from proband peripheral blood leukocytes, following the salting out method [38], quantified by a NanoDrop spectrophotometer ND-1000 (version 3.3; Life Technologies; California, USA) and stored at 4°C until further use. Regarding the proband family, DNA was extracted from the sputum following with the prepIT-L2P kit (DNA Genotek Inc.), quantified by a NanoDrop spectrophotometer ND-1000 (version 3.3; Life Technologies), and submitted to Sanger sequencing.

Total RNA from nasal proband, parents and controls, was extracted with the NZY Total RNA Isolation Kit (NZYTech, Lisbon, Portugal), quantified by NanoDrop spectrophotometer ND-1000 (version 3.3; Life Technologies). The extracted RNA was converted into cDNA, with High-Capacity cDNA Reverse Transcription Kit (Applied Biosystems, California, USA), according to the manufacturer instructions.

### Whole-exome sequencing

Whole-exome sequencing of the proband DNA was performed using the Twist’s Human Comprehensive Exomecapture kit and a NovaSeq sequencer (Illumina, California, USA). For data analysis, a custom validated pipeline, based on the Broad Institute Best Practices, was applied using Burrows-Wheeler Aligner (BWA-MEM) for alignment to the GRCh37 build of the human genome, GATK HaplotypeCaller for variant calling, and Ensembl VEP and GEMINI for variant annotation. Quality control was performed on the resulting FASTQ (FastQC), Binary Alignment MAP (BAM) (QualiMap and samtools), and Variant Call File (VCF) (bcftools) files, aggregated on a quality control report using MultiQC. For CNV calling, we resorted to the VS-CNV (a copy number variation caller) module in the VarSeq software (Golden Helix, Montana, USA).

The VCF contains all the genomic alterations in the coding regions of the patient DNA (i.e., the exome) gathering around 50,000 different alterations. Several filters were applied to detect a potentially pathogenic variation. The first filters used were quality filters, which were used to prevent systematic errors or poor quality genomic regions from interfering with data analysis. Due to the high rate of false discovery in the mutation calling of the HYDIN gene, variants of this gene were thus discarded [39]. Then, variants were analyzed according to the following inclusion criteria: (1) variant frequency below 1% in public databases (including 1000 Genomes Project, NHLBI GO Exome Sequencing Project, and the Genome Aggregation Database (gnomAD)); (2) variant zygosity, with single heterozygote variants being excluded; (3) variant pathogenicity prediction according to computational algorithms (such as Mutation Assessor, Phanter, SIFT, Mutation Taster, Polyphen-2, REVEL, and CAAD); and (4) the function of candidate gene was associated with PCD, cilia, and flagella. Finally, all suspicious variants were inspected on the BAM file through GenomeBrowse version 2.0.2 (Golden Helix, Bozeman, USA) to reject possible false positives.

### Gene expression analysis

The list of used primers is included in Supplementary Table S1. Real-time quantitative PCR (qPCR) was performed in a Bio-Rad CFX96 (Bio-Rad, Hercules, USA), and amplifications prepared with NZY qPCR Green (NZYTech, Lisbon, Portugal), according to manufacturer instructions. Genes *GAPDH* and *ECM7* were used as housekeeping genes. Three technical replicates of the proband, parents, and control samples were performed in each PCR assay. Seven control samples were included. Fold variation of gene expression levels was calculated following a mathematical model using formula 2−ΔΔCt (the Livak method) [40]. Statistical significance was determined using the non-parametric Mann-Whitney statistical test, with the GraphPad Prism software version 9. Significance was set at alpha < 0.05.

### Immunofluorescence

Immunofluorescence analysis of nasal epithelial cells was performed as previously described [12]. Briefly, cell suspensions were spread onto glass slides (STARFROST, Knittel-Glass, Germany), air dried, and stored at −80°C until use. Cells were fixed with 4% paraformaldehyde (20 min, RT) (Merck) in PBS pH 7.4 (Panreac, Barcelona, Spain), permeabilized with 0.2% Triton X-100 (Sigma-Aldrich) (15 min, RT) in PBS, and blocked with 5% non-fat milk (60 min, RT) (Nestlé, Vevey, Switzerland). Cells were then incubated overnight at 4°C with primary antibody rabbit anti-DRC1 (Antibodies on-line, Aachen, Germany) and mouse anti-acetylated α-tubulin (6-11B-1) (Santa Cruz Biotechnology, Texas, USA). The polyclonal antibody anti-DRC1 was produced using a Keyhole limpet hemocyanin conjugated synthetic peptide with sequence 301-400 amino acid derived from human *DRC1* gene (NCBI Gene ID: 92749). For each experiment, a negative control, through the omission of the primary antibody, was included. Anti-rabbit IgG FITC: sc-2359 and anti-mouse IgGk BP-CFL 594 (Santa Cruz Biotechnology) were used as secondary antibodies. Cells were counterstained with Vectashield mounting medium containing 4′,6-diamidino-2-phenylindole (DAPI: Vector Laboratories, California, USA). Results were observed in an epifluorescence microscope (Eclipse E400; Nikon, Tokyo, Japan).

The corrected total cell fluorescence (CTCF) was calculated, according to the formula CTCF = integrated density − (selected cell area × mean fluorescence of background readings). Statistical analysis was performed with the GraphPad Prism software, applying the Mann-Whitney test, with significance levels at alpha < 0.05 (*) (** = *p* value < 0.01; *** = *p* value < 0.001).

## Results

### *DRC1* null variant causes absence of the N-DRC and IDA, and female infertility

The female patient (proband) and her twin sister presented strong clinical signs of PCD, and both have idiopathic infertility.

**Fig. 2:**
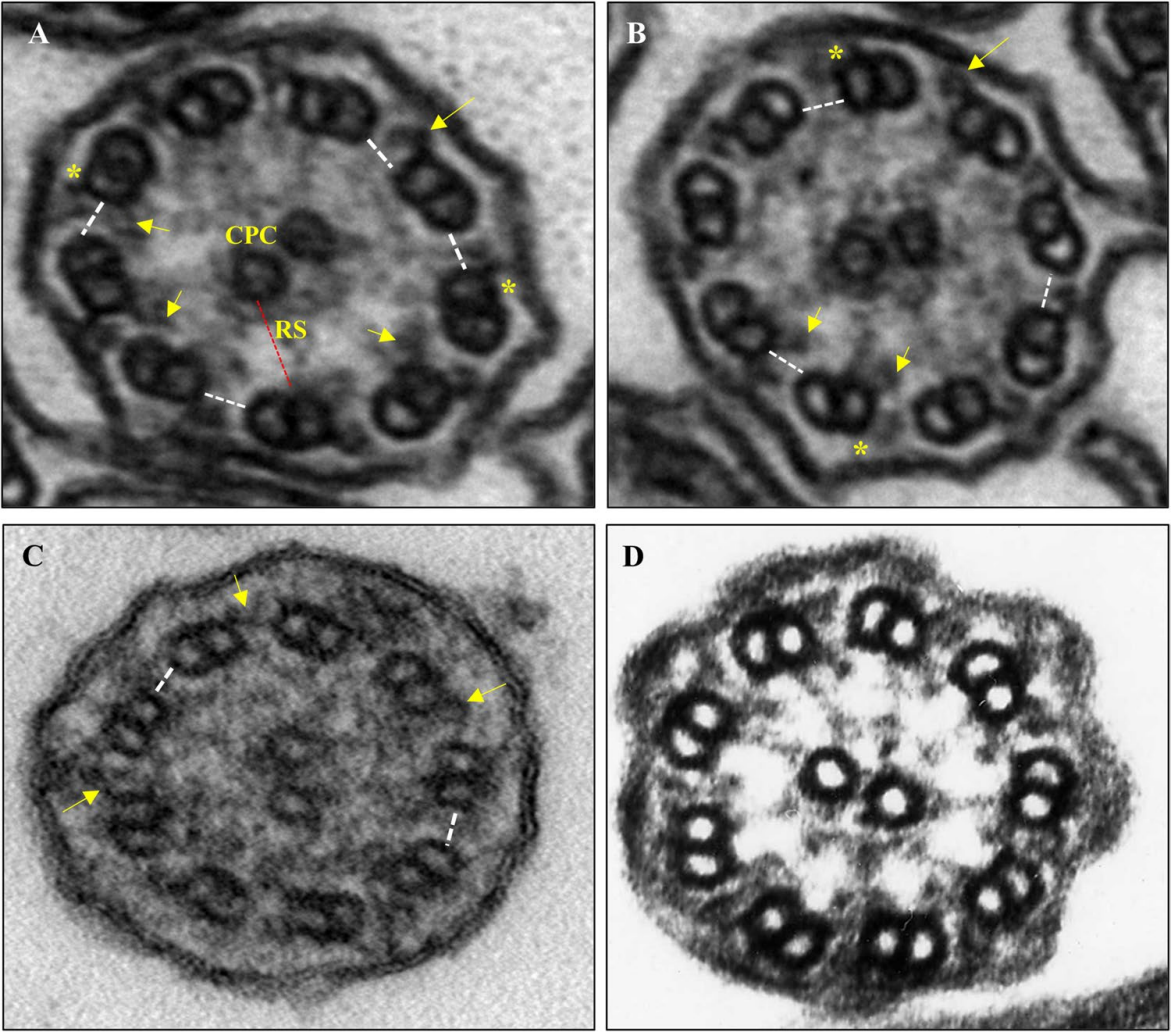
Ultrastructure of cilia axonemes of the proband. Arrows indicate outer and inner dynein arms that serve as the normal pattern. The (*) indicate absence, reduction of dimensions, or little definition of the outer dynein arms. **A** Case with absent, decreased dimensions or poor definition of the outer dynein arms (*n* = 2, pathological >5) and with absence, decreased dimensions, or poor definition of the inner dynein arms (*n* = 6, pathological >7). This is an axoneme with a normal number of both dynein arms. **B** Case with absence, decrease in dimensions, or poor definition of the external dynein arms (*n* = 2, pathological >5) and internal dynein arms (*n* = 7, pathological >7). This is an axoneme with a normal number of both dynein arms although presenting a borderline decrease in the internal dynein arms. **C** Case with absence, decrease in dimensions, or poor definition of the external dynein arms (*n* = 6, pathological >5) and internal dynein arms (*n* = 9, pathological >7). It is an axoneme with decreased number of both dynein arms. In these axonemes, an absence of the N-DRC (white dotted bars) and a normal presence of central pair complex (CPC) and of the radial spokes (RS) was observed. **D** A typical axoneme displaying a normal ultrastructure

The quantitative ultrastructure of nasal cilia axonemes in the proband was performed according to the “international consensus guideline for reporting transmission electron microscopy results in the diagnosis of PCD (BEAT PCD TEM Criteria)” [31]. Our ultrastructural findings revealed the absence of the N-DRC in all high-resolution cross sections and presence of a normal CPC and RS (Fig. 2). Besides, we observed absence of the ODA in 3.8% and absence of the IDA in 22.1% of high-resolution cross sections. The values observed regarding the absence of ODA and IDA are below the international limits of >50% in high-resolution cross sections [31] and thus, are considered to belong to a normal range for DA. The basal bodies were within the normal pattern, well oriented, and aligned. We also quantified the axonemal structural elements indicative of inflammation [41], which gave a value of 18.2%. As this is below the pathologic level of >50%, the observed inflammation marks were not considered to have influence on the structural and functional axoneme changes observed in patient cilia. The variation in ciliary beat axis and ciliary deviation showed a high SD (31.4), which corroborates the diagnosis of PCD in the patient.

Concerning HSVM analysis, proband cilia presented a mean CBF value of 8.97/7.68 (range 0.0-17.24/0.0-22.0), calculated manually and with the CiliarMove software, respectively. These values are below the mean of the reference value (12.75 Hz) (*p* value of 0.032), but still within the normal control range of CBF (7.00-19.00). Regarding CBP, 60% of the patient’s ciliated cells were found to have dyskinetic motions, including 20% stiff (Supplementary Video 1), 20% synchronous with reduced beat amplitude (Supplementary Video 2), and 20% immotile (Supplementary Video 3), and that 40% had normal movements (Supplementary Video 4).

The HSVM analysis in the proband parents revealed in her mother a mean CBF value of 10.72/10.17 (range (5.98-17.33/3.9-15.4), calculated manually/CiliarMove software); and in her father a mean CBF value of 13.26/13.23 (range (8.57-18.75/7.0-19.0) calculated manually/CiliarMove software). In both parents, these CBF values are within the normal range; thus, both are considered normal. Regarding the CBP, her mother showed 77.8% of the ciliated cells with dyskinetic movements, including 5.6% stiff, 72.20% synchronous with reduced beat amplitude, and 22.20% with normal movements. In contrast, the father reveled 69.20% of the ciliated cells with normal movements and 30.8% with dyskinetic movements, specifically a synchronous with reduced beat amplitude pattern.

**Fig. 3:**
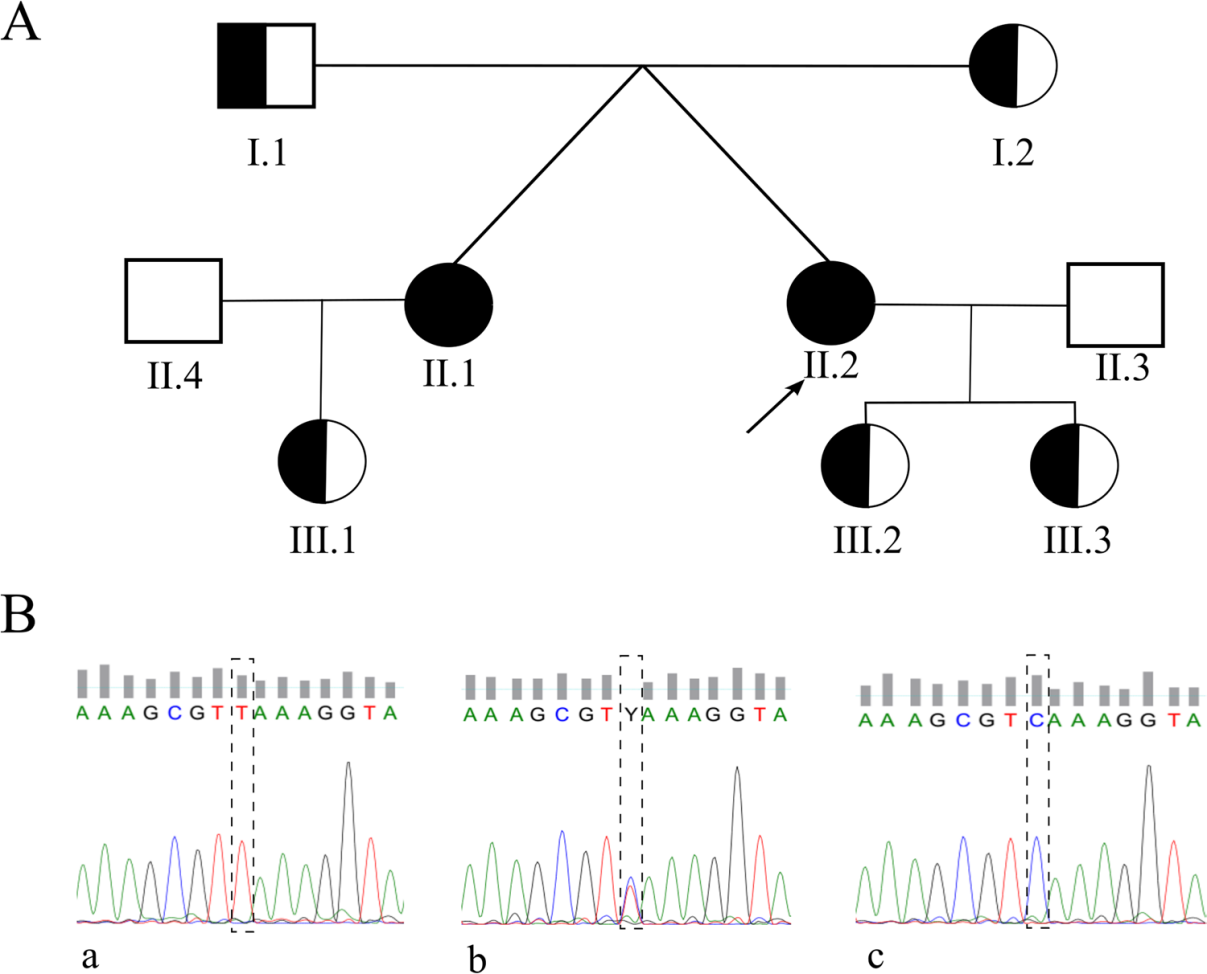
**A** Three-generation family pedigree from the proband (II.2, black arrow). **B** Representative electropherograms from *DRC1* variant NM_145038.4: c.352C>T, obtained after Sanger sequencing, showing the variant in homozygosity (a, present in family members II.1 and II.2), in heterozygosity (b, present in family members I.1, I.2, III.1, III.2, and III.3) and a normal variant (c, family members II. 3 and II.4)

WES analysis of the proband’s DNA revealed a null homozygous variant in the *DRC1* (dynein regulatory complex 1), caused by a substitution of a cytosine for thymine in exon 3 at position 352 (NM_145038.4: c.352C>T) (Fig. 3). This variant has a frequency of 0.04% in the general population (very rare) and is known as a nonsense variant, because this nucleotide substitution creates a premature termination codon (PTC). Consequently, this variant is expected to cause a premature termination of the DRC1 protein at amino acid 118 (p. Gln118*). The gnomAD database classifies this variant as a loss of function variant with high confident score, which gives an additional piece of evidence that this variant is truly loss-of-function. Thus, according to the American College of Medical Genetics and Genomics guidelines [42], this variant is classified as a PVS1 null variant.

Sanger sequencing of sputum DNA from the twin sister revealed the same homozygotic variant found in the proband. As expected, Sanger sequencing of sputum DNA from the parents and the three children confirmed the presence of this variant in heterozygosity. Sanger sequencing of sputum DNA from the husbands was negative.

### The c.352C>T variant in *DRC1* causes reduced mRNA and protein expression

To explore the molecular consequences of this null variant identified in *DRC1*, we analyzed mRNA and protein expression in nasal epithelial cells of the proband in comparison to controls.

**Fig. 4:**
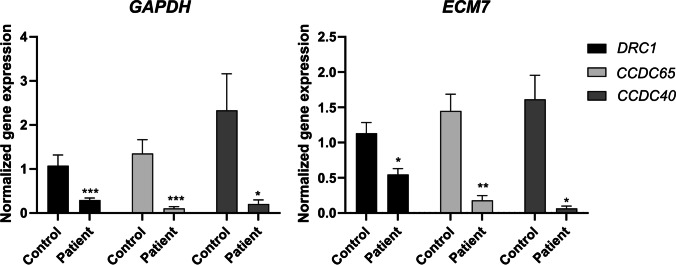
*DRC1*, *CCDC65*, and *CCDC40* mRNA expression levels in nasal cells from the patient in comparison to controls. *GAPDH* (left panel) and *ECM7* (right panel) were used as reference genes. SYBR Green was the fluorescent dye used. Statistical significance was determined using the Mann-Whitney test. * *p* < 0.05, ** *p* < 0.01, and *** *p* < 0.0001

The qPCR analysis revealed that *DRC1* mRNA expression in the proband was significantly reduced in comparison to controls, with a fold change reduction of 0.27 (*p* = 0.0002) for *GAPDH* and 0.45 (*p* = 0.032) for *ECM7* (Fig. 4).

To obtain further knowledge regarding the consequence of the identified variant, we decided to infer its impact on gene expression of related genes; thus, we also performed gene expression analysis for *CCDC65*, *CCDC40*, and *CCDC39*.

Interestingly, mRNA expression of *CCDC65* and *CCDC40* showed a prominent reduction. The *CCDC65* gene showed a fold change reduction of 0.08 (*p* = 0.001) using *GAPDH* as housekeeping gene and 0.15 (*p* = 0.008) with *ECM7* as housekeeping gene. The *CCDC40* gene showed a fold change reduction of 0.05 (*p* = 0.032) with *GAPDH* and 0.14 (*p* = 0.016) with *ECM7* (Fig. 4). Regarding the *CCDC39* gene, no significant differences in gene expression were observed comparing with controls.

**Fig. 5:**
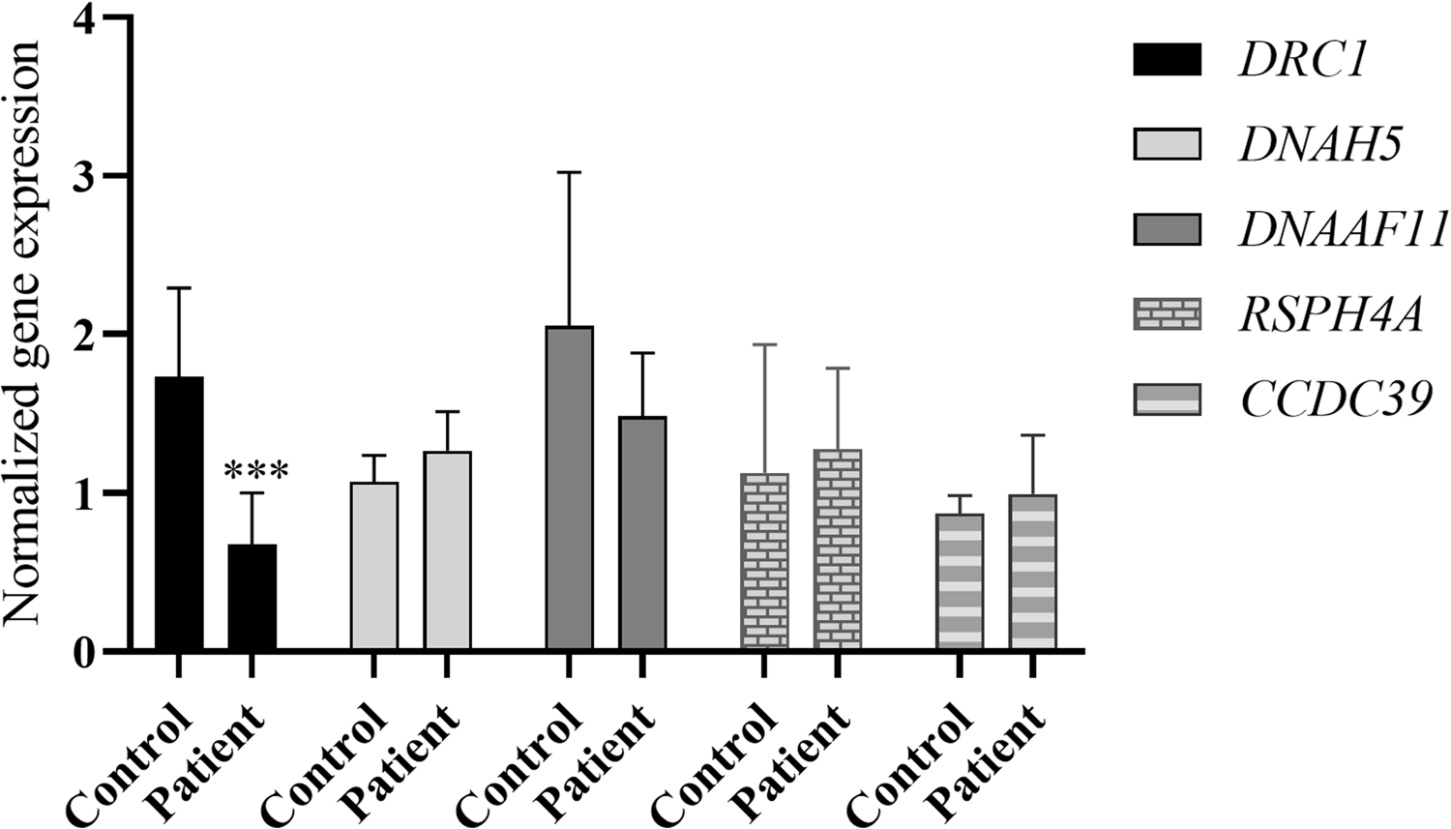
*DRC1*, *DNAH5*, *DNAAF11* (*LRRC6*), *RSPH4A*, and *CCDC39* mRNA expression levels in nasal cells from the patient in comparison to controls. *ECM7* (right panel) were used as reference genes. SYBR Green was the fluorescent dye used. Statistical significance was determined using the Mann-Whitney test. * *p* < 0.05, ** *p* < 0.01, and *** *p* < 0.0001

To infer if this observed reduction in gene expression is indeed causal and not caused by an eventual decrease in the multiciliated cells, we performed gene expression analysis for other ciliary genes not directly associated with the *DRC1* gene, namely *DNAH5*, *DNAAF11* (*LRRC6*), and *RSPH4A*. *DNAH5* is a well-known gene in PCD genetics and encodes a heavy chain of the ODA [43]. *DNAAF11* is an axonemal dynein preassembly gene [44] and *RSPH4A* codes for an RS-head component [45]. We observed no significant differences in gene expression comparing with controls (Fig. 5).

**Fig. 6:**
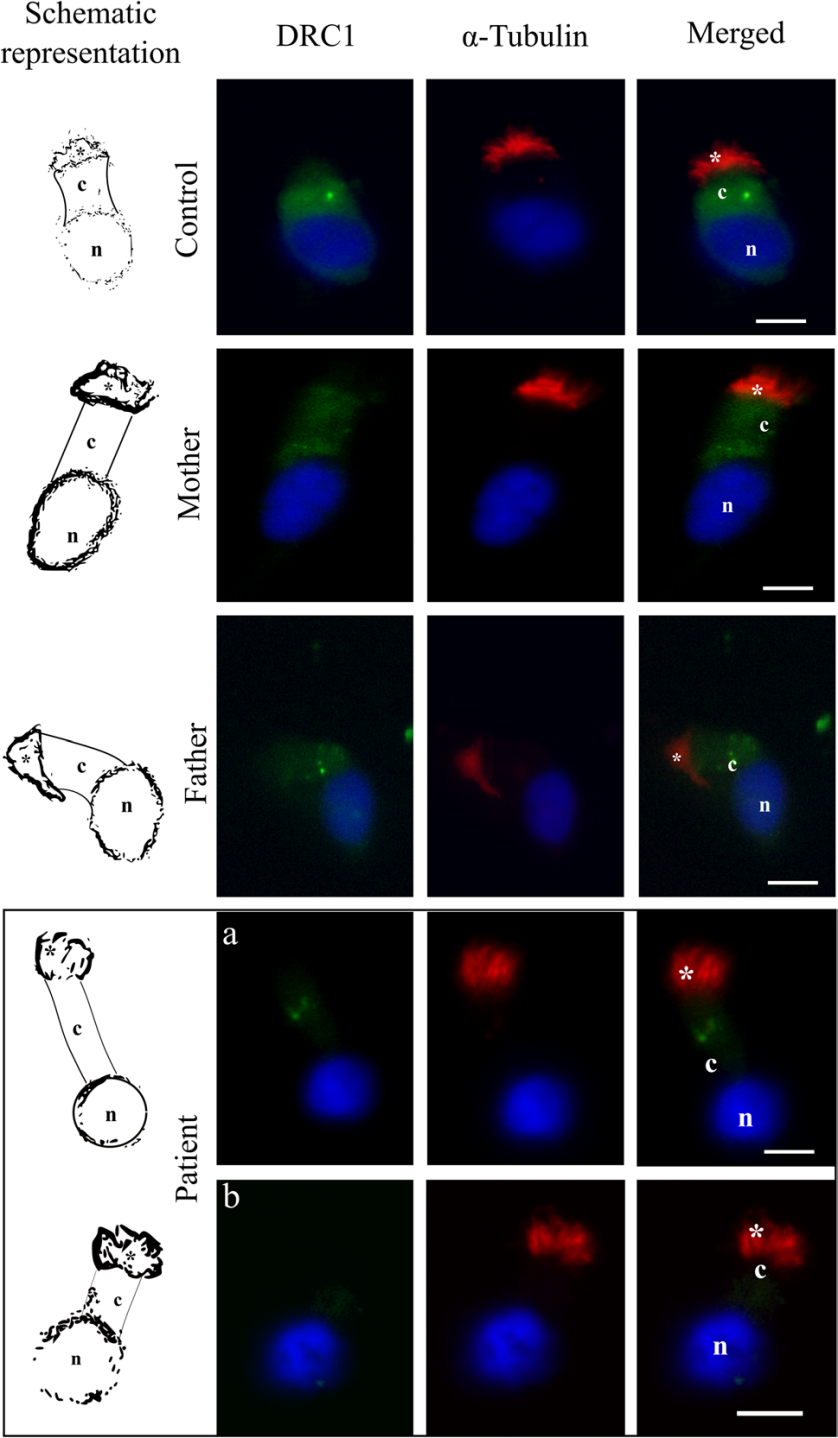
Immunocytochemical detection of DRC1 (green) and of axoneme-specific acetylated α-tubulin (red), with merged images, in nasal cilia cells of a control, the patient mother and father and the patient. In both controls, the DRC1 staining appears in the cytoplasm. Regarding patient cells, an example of cilia with a weak staining (a) and other without staining (b) are shown. Nuclei stained with DAPI (blue). * cilia, *c* cytoplasm, *n* nucleus. Scale bars: 5 μm

Relatively to protein expression analysis by immunofluorescence, our work was the first described DRC1 protein expression in nasal cells. Using an antibody for DRC1, we observed an intense staining in the cytoplasm of control nasal cells (Fig. 6). In proband nasal cells, a very weak cytoplasmic staining in some ciliated cells was observed while others did not present any staining (Fig. 6). Fluorescence quantification indicated that, on average, the intensity of the fluorescence signal is 0.06 times lower compared to controls (*p* < 0.0001). This indicates a reduction in protein expression, which agrees with the type of variant and the results from mRNA expression analysis. Regarding parents, we observed an intense staining in the cytoplasm as in control nasal cells.

## Discussion

Here we firstly report a *DRC1* null variant associated with female infertility in a patient with a clinical diagnosis of PCD and idiopathic infertility. The PCD diagnosis was confirmed by different methods, namely by HSVM, ultrastructural analysis, and genetic screening.

The proband and her sister suffer, since infancy, of recurrent otitis media, respiratory infections, and rhinosinusitis, and both had a diagnosis of idiopathic infertility. Both only achieve a successful pregnancy after IVF. Interestingly, the Sanger sequence screen in the twin sister evidenced the same homozygous variant. As expected, parents and children (proband daughters and niece) evidenced the variant, but in heterozygosity. Unfortunately, because the sister and her family live outside of the continent, it was not possible to obtain a nasal brush and thus conduct further analysis.

Two recent works have also reported anomalies in *DRC1* as a cause for PCD in men with multiple morphological abnormalities of the sperm flagella (MMAF) [46, 47], but so far, no female cases of infertility were associated with *DRC1*. Given the *DRC1* proposed role and the fact that both twins present the same null variant in the *DRC1* gene, we propose that this variant is the cause of PCD and infertility in the twins. Infertility is most likely caused by cilia defects in the fallopian tubes, which are expected to cause dyskinetic ciliary beating, like that seen in respiratory cilia, making embryo propulsion into the uterine cavity for implantation more difficult.

A link between fertility status, ultrastructural findings observed by TEM, and the genotype was already proposed [13], with mutations in the genes *CCDC39*, *CCDC39*, *DNAAF1*, *DNAAF2*, *DNAAF4*, *HYDIN*, *LRRC6*, *MCIDAS*, *NME8*, *ODAD3*, and *RSPH3* causing infertility in all studied women [13, 48–50]. Nevertheless, this list is much lower in women PCD-related fertility comparing to men, highlighting the need for additional research on female PCD infertility.

The results from qPCR analysis revealed a reduction in mRNA expression of *DRC1*, *DRC2*, and *CCDC40*.

DRC1 and DRC2 proteins seem to form a subcomplex and to interact intimately with each other, as shown in *Chlamydomonas* mutants, where absence of DRC1 or DRC2 caused reduced densities for the entire N-DRC base plate, including the DRC4 subunit [24]. Our results from gene expression thus agree with previous works and support the hypothesis that DRC1 and DRC2 form a subcomplex.

CDC40 and CCDC39 were previously proposed to govern the assembly of the N-DRC and IDA complexes [51, 52], so we thought that both could be affected by the *DRC1* null variant. However, we only observed a significant mRNA expression reduction for *CCDC40* and did not detect any alteration in *CCDC39* expression. Previous study on *Chlamydomonas* mutants suggested that the DRC1 function is not dependent on the assembly of CCDC39 [23], which could justify our findings and support the hypothesis that DRC1 and CCDC39 are independent.

*CCDC40* were proposed as a likely gene to cause infertility [13]; thus, the mRNA expression reduction here observed in this may also contribute to the infertility of our proband.

None of the previous studies that identified DRC1 variants performed gene expression analysis of DRC1 or of the other N-DRC-related genes. Therefore, we cannot discuss whether the observed morphologic data could be due to a distinct mRNA expression pattern.

To infer if the observed reduction in gene expression observed in *DRC1*, *DRC2*, and *CCDC40* is caused by an eventual decrease in the multiciliated cells, we performed gene expression analysis in other ciliary genes, namely *DNAH5*, *DNAAF11*, and *RSPH4A*. However, no significant differences in gene expression were observed comparing with controls (Fig. 5), which suggests that the reduced expression of *DRC1*, *CCDC65*, and *CCDC40* are not caused by a decrease in multiciliated cells.

Further studies are needed to further explore the association of *DRC1* null variants and *DRC2* and *CCDC40* mRNA reduction. Now, we could only speculate hypotheses to justify it. One possibility is the fact that we have only analyzed the coding region, and thus we could not rule out the hypothesis of the existence of mutations in the non-coding regions of those genes being associated with this mRNA reduction. A previous work has shown that apparently neutral polymorphic variants can modulate the clinical phenotype [53], and likely could interfere with mRNA expression. Another, possible explanation is that it could be due to a phenomenon similar to transcriptional adaptation. Although this phenomenon of translation adaptation has been mostly associated with an upregulation of other related genes, it is still largely unknown. For instance, other authors have studied this event and found that reducing transcription of the mutant Fermt2 gene in Fermt2 knockout cells led to a decrease in Fermt1 mRNA levels [54].

Our ultrastructural findings, although partial, are in accordance with the observations in *Chlamydomonas* mutant for *DRC1*, where authors observed that disruption of the *DRC1* subunit resulted in assembly defects in N-DRC and IDA [23]. Other cases involving *DRC1* variants, but not infertility, have been reported, primarily in Asia, where *DRC1* variants are common. In non-Asian populations, the prevalence of *DRC1* variants is rare [55]. A biallelic deletion of *DRC1*, spanning 27,748 bp, including exons 1-4, was detected by three independent studies. First is by Morimoto et al., in 2019 [56], then by Keicho et al., in 2020 [57], and subsequently by Takeuchi et al., also in 2020 [58]. In those three independent studies, the ciliary ultrastructural findings were heterogeneous, with predominance of axonemal microtubular disorganization. In India, a homozygous nonsense *DRC1* variant, resulting in a stop codon occurring in exon 10, was also observed (NM_145038: c.1205G>A; p.Trp402*) [59]. In this study, TEM was not performed; thus, we could not compare the findings, but HSVM analysis also revealed dyskinetic movement in most cilia [59]. In Tunisia, a frameshift *DRC1* variant was observed in exon 2 (c.2012_213del; p. Ser70Argfs*11). Here neither TEM nor HSVM was performed [60]. A homozygous nonsense *DRC1* variant (c.2056A>T), also causing a premature stop of translation (p.Lys686*), was described in an Austrian patient with Turkish ancestry, and, in the same work, two Swedish families presented PCD with the same *DRC1* variant here reported [23]. The Swedish families with the same *DRC1* variant here identified (NM_145038.4: c.352C>T, p. Gln118*) also had a reduced CBF and a CBP with a reduced ciliary amplitude and stiff movements compared to wild-type cilia. However, while we observed N-DRC anomalies associated with female infertility, Swedish patients did not report infertility issues [23]. In the other studies, none of the patients with a *DRC1* variant had situs inversus, as in our patient, which suggests that *DRC1* somehow do not affect the nodal cilia.

The variety of findings regarding ultrastructural (microtubular disorganization, absent N-DRC, absent N-DRC+IDA, absent CPC) and clinical findings (infertility, fertility) is not newer to PCD. Other authors have also reported a distinct spectrum of ultrastructural defects in individuals carrying the same mutation in *CCDC103* gene [61]. This is another example of the wide phenotypic variability observed in PCD. Modifier genes, epigenetic changes, incomplete penetrance, post-translational modifications, and many other steps that lie between gene transcription and protein expression, which are still largely unknown particularly in PCD, may justify the observed variability.

Here we report, for the first time, a *DRC1* variant associated with female infertility in the Iberian Peninsula, and firstly report the immunolocalization of DRC1 in human nasal cells, both in healthy individuals and in a PCD patient carrying a *DRC1*-null variant. As expected, the immunofluorescence analysis showed a significant staining reduction of DRC1 protein in patient nasal cells. In control nasal cells and proband’s parents, we observed an intense staining in the cytoplasm, about 16.78 times higher than the staining observed in our PCD patient. The observed staining of DRC1 in the cytoplasm was not expected. As DRC1 is a member of the N-DCR, a component of the axoneme, it was therefore expected to be detected along the cilia axoneme. This outcome can have different explanations. First, all proteins necessary for ciliary development and function are synthetized in the cytoplasm and then transported into the cilium compartment. During transport, many post-translational modifications, including glycosylation, lipid acylation, and prenylation, occur, particularly at the Golgi [62]. It is thus possible that the antibody used could only detect the unprocessed form of DRC1 in the cytoplasm. The antibody used was the only available on the market at the time of the study and, therefore, we could not test a second antibody to infer if another antibody, produced with a different epitope, could raise a different outcome. This should be tested in the future, as new antibodies become available.

The fact that in our ultrastructural analysis we have observed a heterogeneous pattern, as well as observed in immunofluorescence analysis (by detected some minor staining in some cells of our patient and others with no staining at all), may be related with an nonsense-mediated mRNA decay (NMD) evasion phenomenon, in which some premature termination codons (PTCs) containing transcripts are still translated into proteins [63]. In proband parents, no change in staining was observed, suggesting that, for development of a detrimental effect, both alleles need to be mutated, as expected for an autosomal recessive disease.

We are aware that our study has limitations due to the small number of participants and the lack of animal models used to validate our findings. Our report can only be seen as a starting point to further studies that enlighten about the interaction of *DRC1* with other genes. To further explore the pathogenicity of this variant and its gene interactions and effects on fertility, studies with animal models are needed.

## Conclusions

Together, this work demonstrates strong evidence for the association of *DRC1* and its pathogenic variant c.352C>T with the PCD phenotype and female infertility. As far as we know, this study is the first to report two infertile PCD women carrying a *DRC1* variant. Furthermore, we were also the first to report *DRC1* mRNA and protein expression studies in human nasal cells under normal and pathologic conditions. We also evidenced an interaction among *DRC1*, *DRC2*, and *CCDC40* genes, being thus critical to explore this finding in future studies, as it may be an explanation for the clinical heterogeneity presented by patients with PCD. This work also emphasizes the importance of all PCD specialists being aware of fertility issues, and the fact that fertility counseling should be included in standard PCD patient care.

To sum up, our findings provided another element to the challenging genetic study of PCD and may ultimately aid in a better understanding of the pathophysiology of PCD and infertility.

## Supplementary information

Supplemental video 1 Cilia showing a stiff movement. (AVI 15186 kb)

Supplemental video 2 Cilia exhibiting reduced beating amplitude (AVI 8143 kb)

Supplemental video 3 Immotile cilia movement. (AVI 5465 kb)

Supplemental video 4 Normal cilia movement. (AVI 9571 kb)

Supplemental Table S1 List of primers used in this study. (DOCX 16 kb)

## Data Availability

The authors confirm that the data supporting the findings of this study are available within the article and its supplementary materials. This article does not include any specific dataset necessary to interpret, replicate, and build upon the findings reported in the article.
