# Supplementary material for: Characterization of a DRC1 null variant associated with primary ciliary dyskinesia and female infertility
Source: J Assist Reprod Genet. 2023 Mar 1;40(4):765–78. doi: 10.1007/s10815-023-02755-6 (PMC10224902; doi:10.1007/s10815-023-02755-6)
Supplement: Supplementary file 5 — List of primers used in this study. (DOCX 16 kb) [file 10815_2023_2755_MOESM5_ESM.docx]

| **Gene** | **Primer sequence (5' -> 3')** | **NCBI Reference Transcript** | **Length** | **Tm** | **GC%** |
| --- | --- | --- | --- | --- | --- |
| GAPDH-F | A​G​G​T​C​G​G​A​G​T​C​A​A​C​G​G​A​T​T​T​ | NM_001289745 | 20 | 64 | 50 |
| GAPDH-R | TGGAATTTGCCATGGGTGGA |  | 20 | 64 | 50 |
| EMC7-F | ATGAGACGGGAAATGGAGCA | NM_020154 | 20 | 59.09 | 50.00 |
| EMC7-R | CCAGTGTTGCCGTGTTTGTG |  | 20 | 60.53 | 55.00 |
| DRC1-F | GAGCCTTTGATGTGGACAGG | NM_145038 | 20 | 58.54 | 55.00 |
| DRC1-R | TCTGTGTGGCGGACTTCTG |  | 19 | 59.63 | 57.89 |
| CCDC65-F | TGAAAGCAAGCTGGAGTTCC | NM_001286957 | 20 | 58.39 | 50.00 |
| CCDC65-R | CAGCCTTTCGATCCTCTGTG |  | 20 | 58.35 | 55.00 |
| CCDC39-F | AGCAAGGATAAAGCAGGAAACG | NM_181426 | 22 | 59.25 | 45.45 |
| CCDC39-R | TCATGGTCCTGATATGCCGT |  | 20 | 58.58 | 50.00 |
| CCDC40-F | TTAGGCCCGTCGGAGCAAATGG | NM_017950 | 22 | 65.15 | 59.09 |
| CCDC40-R | ACTGGCTCCTGCGAGACGAACT |  | 22 | 65.87 | 59.09 |
| RSPH4A-F | GCATATTCTCTCTCAGGGTCG | NM_001010892 | 21 | 57.70 | 52.38 |
| RSPH4A-R | TTGGTGTCAAAAGAGGAAGCC |  | 21 | 58.69 | 47.62 |
| DNAH5-F | GCTGGATGACGGTGCAAAACCT | NM_001369.3 | 22 | 64 | 54.50 |
| DNAH5-R | AACCGCTTGGCTTCCTTGGG |  | 20 | 63 | 60.00 |
| DNAAF11 (LRRC6)-F | GCCATGGGCTGGATCAC | NM_012472.6 | 17 | 57.68 | 64.70 |
| DNAAF11 (LRRC6)-R | TGCTGATGCAACGAGAGTTC |  | 20 | 58.57 | 50.00 |

Supplemental Table 1: List of the PCR primers used in this study.

CG%- CG content of the primer, F- Forward Primer, R- Reverse Primer, Tm- Temperature Melting of the primer.
